# Neonatal Bacillus Calmette‐Guérin Vaccination Decreases Eczema Incidence at 5 years: The MIS BAIR Randomised Controlled Trial

**DOI:** 10.1111/all.16677

**Published:** 2025-07-31

**Authors:** Laure F. Pittet, Nicole L. Messina, Kate L. Francis, Susan Donath, Katie L. Flanagan, Emily K. Forbes, Kaya Gardiner, Roderic J. Phillips, Anne‐Louise Ponsonby, Roy Robins‐Browne, Frank Shann, Mike South, Peter Vuillermin, Dan Casalaz, Nigel Curtis, On the behalf of the Melbourne Infant Study: BCG for Allergy and Infection Reduction (MIS BAIR) Group

**Affiliations:** ^1^ Infectious Diseases Group Murdoch Children’s Research Institute Parkville Victoria Australia; ^2^ Department of Paediatrics The University of Melbourne Parkville Victoria Australia; ^3^ Immunology, Vaccinology, Rheumatology and Infectious Diseases Unit, Faculty of Medicine Geneva University Hospitals and University of Geneva Geneva Switzerland; ^4^ Clinical Epidemiology and Biostatistics Unit Murdoch Children’s Research Institute Parkville Victoria Australia; ^5^ School of Health Sciences University of Tasmania Hobart Tasmania Australia; ^6^ School of Health and Biomedical Science RMIT University Melbourne Victoria Australia; ^7^ Department of Immunology and Pathology Monash University Melbourne Victoria Australia; ^8^ Department of Research Operations The Royal Children’s Hospital Melbourne Parkville Victoria Australia; ^9^ Dermatology Unit The Royal Children’s Hospital Melbourne Parkville Victoria Australia; ^10^ Population Allergy Murdoch Children’s Research Institute Parkville Victoria Australia; ^11^ The Florey Institute for Neuroscience and Mental Health The University of Melbourne Melbourne Victoria Australia; ^12^ Department of Microbiology and Immunology Peter Doherty Institute for Infection and Immunity, The University of Melbourne Melbourne Victoria Australia; ^13^ Department of General Medicine The Royal Children’s Hospital Melbourne Parkville Victoria Australia; ^14^ School of Medicine Deakin University Geelong Victoria Australia; ^15^ Child Health Research Unit Barwon Health Geelong Victoria Australia; ^16^ Neonatal Intensive Care Unit Mercy Hospital for Women Heidelberg Victoria Australia; ^17^ Infectious Diseases The Royal Children’s Hospital Melbourne Parkville Victoria Australia

**Keywords:** atopic dermatitis, prevention, vaccines

To the Editor,

Eczema significantly impacts the health and quality of life of children and their families. We previously reported that neonatal vaccination with Bacille Calmette‐Guérin (BCG) reduces the incidence of eczema by 12% (95% confidence intervals (CI) −0.4 to 26%) in the first year of life [1]. We now report the five‐year follow‐up results.

The *Melbourne Infant Study: BCG for Allergy and Infection Reduction* (MIS BAIR) trial was designed to investigate whether neonatal BCG vaccination reduces the incidence of eczema, allergy, asthma, and infections in the first 5 years of life [2]. In this phase 3, multicentre, randomised controlled trial, 1272 infants were randomised at birth to the BCG vaccination or control (no BCG) group in Victoria, Australia. Infants randomised to the intervention group received 0.05 mL of the BCG‐Denmark vaccine (Statens Serum Institute; *Mycobacterium bovis*, Danish strain 1331) intradermally in the left deltoid region before 10 days of age. Participants were followed up for 5 years with online questionnaires and clinic visits. ClinicalTrial.gov: NCT01906853.

The primary analysis was the cumulative incidence of eczema by 5 years, using William's UK diagnostic criteria from questionnaire data [3]. Binary regression adjusted for stratification factors was used to calculate adjusted risk differences (aRD) with 95% CI. Multiple imputation models were used to handle missing data. Outcome definitions, analyses, and subgroup analyses are detailed in the statistical analysis plan (available on request). Stata v.18.0 was used for all analyses.

Between August 2013 and September 2016, 637 infants were allocated to the BCG group and 635 to the control group (Figure E1, Table E1). By 5 years of age, 37.1% of children in the BCG group developed eczema compared to 45.3% in the control group (adjusted risk difference [aRD] −8.2%, 95% CI −14.1% to −2.2%; 18.1% relative risk reduction; number‐needed‐to‐treat 12; Figure 1). The protective effect of BCG was found with different definitions of eczema (Table E2), including reported use of topical steroid (aRD −8.0%, 95% CI −14.9% to −1.2%; 11% relative risk reduction). Subgroup analyses revealed no evidence of interaction (Figure E2). Children born to two atopic parents were more likely to have eczema. In this high‐risk group, the cumulative incidence of eczema was lower in the BCG group (43%) compared with the control group (54%; aRD −11.6 percentage points, 95% CI −22.7 to −0.5; 21.3% relative risk reduction; number‐needed‐to‐treat 9). Further details are provided in the Appendix S1.

**FIGURE 1:**
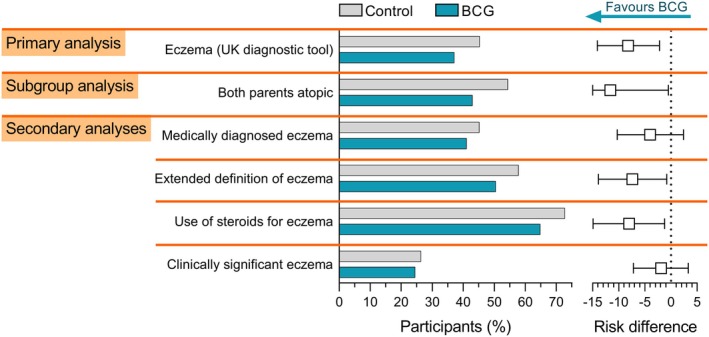
Difference in incidence and severity of eczema between BCG and control groups. Left: Bars represent the proportion of participants fulfilling the criteria for the primary and secondary analyses, with error bars depicting 95% confidence intervals (CI). Right: Squares represent adjusted risk differences, with error bars depicting 95% CI. Further details on outcomes definition can be found in the appendix or in the statistical analysis plan (available on request).

These results provide strong evidence that neonatal vaccination with BCG‐Denmark offers a clinically significant protective effect against eczema that persists into childhood. These findings add to previous research suggesting that BCG vaccination has protective effects against atopic diseases [4], particularly eczema [5]. In a meta‐analysis involving over 5600 children in three RCTs (including the MIS BAIR trial), early‐life BCG vaccination reduced the risk of eczema by 11% in the first 13 to 18 months of life [5]. However, at 1 year of age, it is difficult to predict which participants will have transient versus persisting eczema. Our trial found an even greater relative reduction (18%) in eczema over an extended five‐year follow‐up. This further supports the hypothesis that early microbial exposure, represented by the live‐attenuated BCG vaccine, modulates the immune system to reduce susceptibility to atopic diseases [6]. Early‐life immunomodulators should be further investigated as a preventive strategy for atopic diseases, as they may have important implications for public health strategies to reduce the social and economic burden of atopic diseases. Further research is also needed to elucidate the immunological mechanisms by which BCG vaccination confers protection against atopic diseases and to explore its effects on other atopic outcomes.

## Author Contributions

N.C. was the lead investigator and responsible for study conception, design, and funding acquisition. N.C. and S.D. developed the final scientific protocol and ethics application, and all other authors provided critical evaluation and revision. K.G. coordinated, and N.C., D.C., N.L.M., and P.V. were involved in implementation. L.F.P. developed, and N.C., R.P., K.G., N.L.M., A.L.P., S.D., F.S., R.R.‐B., K.L.Fr., and K.L.Fl. contributed to the statistical analysis plan. K.L.Fr. led, and L.F.P. and S.D. contributed to statistical analysis. L.F.P. drafted the manuscript, coordinated manuscript preparation, and revision. All authors provided critical evaluation and revision of the manuscript.

## Conflicts of Interest

The authors declare no conflicts of interest.

## Supporting information


Appendix S1.

## Data Availability

The data that support the findings of this study are available from the corresponding author upon reasonable request.

## References

[1] L. F. Pittet , N. L. Messina , K. Gardiner , et al., “Prevention of Infant Eczema by Neonatal Bacillus Calmette‐Guerin Vaccination: The MIS BAIR Randomized Controlled Trial,” Allergy 77, no. 3 (2022): 956–965. 34309859 10.1111/all.15022

[2] N. L. Messina , K. Gardiner , S. Donath , et al., “Study Protocol for the Melbourne Infant Study: BCG for Allergy and Infection Reduction (MIS BAIR), a Randomised Controlled Trial to Determine the Non‐Specific Effects of Neonatal BCG Vaccination in a Low‐Mortality Setting,” BMJ Open 9, no. 12 (2019): e032844. 10.1136/bmjopen-2019-032844 PMC6924750 31843845

[3] H. C. Williams , P. G. Burney , R. J. Hay , et al., “The U.K. Working Party's Diagnostic Criteria for Atopic Dermatitis. I. Derivation of a Minimum Set of Discriminators for Atopic Dermatitis,” British Journal of Dermatology 131, no. 3 (1994): 383–396. 7918015 10.1111/j.1365-2133.1994.tb08530.x

[4] S. Navaratna , M. J. Estcourt , J. Burgess , et al., “Childhood Vaccination and Allergy: A Systematic Review and Meta‐Analysis,” Allergy 76, no. 7 (2021): 2135–2152. 33569761 10.1111/all.14771

[5] L. F. Pittet , L. M. Thostesen , P. Aaby , P. E. Kofoed , N. Curtis , and C. S. Benn , “Neonatal BCG Vaccination to Prevent Infant Eczema: A Systematic Review and Meta‐Analysis,” Dermatitis 33, no. 6S (2022): S3–S16. 36125788 10.1097/DER.0000000000000945 PMC9674447

[6] B. Freyne , A. Marchant , and N. Curtis , “BCG‐Associated Heterologous Immunity, a Historical Perspective: Experimental Models and Immunological Mechanisms,” Transactions of the Royal Society of Tropical Medicine and Hygiene 109, no. 1 (2015): 46–51. 25573108 10.1093/trstmh/tru196

