# Supplementary material for: Neonatal Bacillus Calmette‐Guérin Vaccination Decreases Eczema Incidence at 5 years: The MIS BAIR Randomised Controlled Trial
Source: Allergy. 2025 Jul 31;80(12):3444–6. doi: 10.1111/all.16677 (PMC12666751; doi:10.1111/all.16677)
Supplement: Supplementary file 1 — Appendix S1. [file ALL-80-3444-s001.docx]

# Appendix

**Neonatal BCG vaccination decreases eczema incidence at 5 years:
the MIS BAIR randomised controlled trial**

Laure F. Pittet, Nicole L. Messina, Kate L Francis, Susan Donath, Katie L. Flanagan, Emily K. Forbes, Kaya Gardiner, Rod Phillips, Anne-Louise Ponsonby, Roy Robins-Browne, Frank Shann, Mike South, Peter Vuillermin, Dan Casalaz, Nigel Curtis, on behalf of the Melbourne Infant Study: BCG for Allergy and Infection Reduction (MIS BAIR) Group.

## Supplement to methods

The trial involved Part 1 (from birth to one year of age) and Part 2 (from one to five years of age). At 1 year of age, participants were asked to re-consent if they wished to continue into Part 2 of the study. The randomisation schedule was developed by an independent statistician, using a web-based computerised system (REDCap®). Random permuted blocks of varying size ensured concealment of allocation, in a 1:1 ratio, stratified by mode of delivery (vaginal vs. caesarean), plurality of birth (singleton vs. twin) and recruitment hospital (Melbourne vs Werribee vs Geelong). Because BCG causes a visible scar, families were aware of the group allocation, but the statisticians and the research nurses doing the study visits remained blinded. Parents were instructed not to reveal their child’s allocation group to the study staff, and to cover the left upper arm of the participant with a bandage during each study visit to obscure any potential scar.

The primary analysis was the cumulative incidence of eczema by five years of age, defined using William’s UK diagnostic tool from questionnaire data. Outcome definitions are detailed in the statistical analysis plan (available on request). Secondary analyses included (i) parent report of medically diagnosed eczema, (ii) extended definition of eczema (using William’s UK diagnostic tool or parent report of medically diagnosed eczema), (iii) parent-reported use of topical steroids, (iv) clinically significant eczema (defined as fulfilling William’s UK diagnostic criteria, being medically diagnosed and treated with steroids), (v) age of onset of eczema, and (vi) eczema severity.

A catch-up questionnaire was sent to participants who had missing data for William’s UK diagnostic criteria or the question about the medical diagnosis of eczema. As participants were then aged seven to ten years old, the answers were only used to identify those who had not had the outcome. This means that only ‘no’ responses (i.e. indicating the participant had never had the outcome) were considered as valid data. ‘Yes’ responses were not considered, as eczema could have started after the study had ended. A sensitivity analysis was done, disregarding the data collected through the catch-up questionnaire.

Multiple imputation (MI) by chained equations was conducted to handle missing data, as this approach to MI can handle multivariable missingness. A single imputation model with all outcome variables included would not converge therefore, an individual imputation model was estimated for the primary analysis and secondary analyses.

## Supplement to results

A worldwide shortage of BCG meant enrolment had to cease prematurely. At the 1-year follow-up, 1027 consented to continue into Part 2 (81%, Figure E1). By five years, complete outcome data for the primary analysis were available for 755/1027 (74%) of these participants, their characteristics are presented in Table E1. The 1-year visit was attended by 1094/1272 (86%) participants at a median age of 1.1 (IQR 1.1 to 1.2 years); the 5-year visit was attended by 435/1027 (42%) participants at a median age of 5.3 (IQR 5.1 to 5.8) years.

Results are presented in Table E2, Figure E2 (subgroup analyses) and Figure E3 (age of onset).

### **Table E1**: Participants characteristics and exposures during the first year of life, by randomisation group, as well as with and without missing data for primary analysis of eczema at 5 years

|  |  | All participants | BCG | No BCG | Not missing | Missing |
| --- | --- | --- | --- | --- | --- | --- |
|  | N | n=1272 | n=637 | n=635 | N=854 | N=418 |
| **Infant factors** |  |  |  |  |  |  |
| Sex, female | 1272 | 630 (49.5%) | 318 (49.9%) | 312 (49.1%) | 436 (51%) | 206 (49%) |
| Birth weight, kg | 1272 | 3.4 (0.5) | 3.4 (0.5) | 3.4 (0.5) | 3.4 (0.5) | 3.4 (0.5) |
| Gestational age at birth, weeks | 1272 | 39.3 (1.4) | 39.4 (1.41) | 39.2 (1.4) | 39.4 (1.4) | 39.1 (1.5) |
| Twin pregnancy | 1272 | 21 (1.7%) | 11 (1.7%) | 10 (1.6%) | 11 (1%) | 10 (2%) |
| Vaginal delivery | 1272 | 812 (63.8%) | 406 (63.7%) | 406 (63.9%) | 559 (66%) | 253 (61%) |
| Infant ethnicity | 1272 |  |  |  |  |  |
| Caucasian (3 to 4 grandparents Caucasian) |  | 949 (74.6%) | 474 (74.4%) | 475 (74.8%) | 643 (75%) | 306 (73%) |
| Asian (3 to 4 grandparents Asian) |  | 82 (6.4%) | 43 (6.8%) | 39 (6.1%) | 61 (7%) | 21 (5%) |
| Mixed Caucasian and Asian |  | 62 (4.9%) | 32 (5.0%) | 30 (4.7%) | 47 (6%) | 15 (4%) |
| Other |  | 179 (14.1%) | 88 (13.8%) | 91 (14.3%) | 103 (12%) | 76 (18%) |
| Season of birth | 1272 |  |  |  |  |  |
| Summer |  | 286 (22.5%) | 149 (23.4%) | 137 (21.6%) | 191 (22%) | 95 (23%) |
| Autumn |  | 362 (28.5%) | 177 (27.8%) | 185 (29.1%) | 242 (28%) | 120 (29%) |
| Winter |  | 314 (24.7%) | 158 (24.8%) | 156 (24.6%) | 218 (26%) | 96 (23%) |
| Spring |  | 310 (24.4%) | 153 (24.0%) | 157 (24.7%) | 203 (24%) | 107 (26%) |
| **Maternal factors** |  |  |  |  |  |  |
| Age at delivery, years | 1271 | 32.6 (4.8) | 32.6 (4.8) | 32.7 (4.7) | 33.0 (4.5) | 31.9 (5.2) |
| History of BCG vaccination | 1206 | 318 (26.4%) | 159 (26.3%) | 159 (26.5%) | 213 (26%) | 105 (27%) |
| Maternal education | 1269 |  |  |  |  |  |
| No education / up to year 10 |  | 75 (5.9%) | 41 (6.5%) | 34 (5.4%) | 28 (3%) | 47 (11%) |
| Year 12 / trade |  | 340 (26.8%) | 165 (26.0%) | 175 (27.6%) | 202 (24%) | 138 (33%) |
| University |  | 854 (67.3%) | 428 (67.5%) | 426 (67.1%) | 622 (73%) | 232 (56%) |
| **Familial and environmental factors at birth** |  |  |  |  |  |  |
| Number of household habitants | 1272 | 2.9 (1.1) | 2.9 (1.2) | 2.9 (1.1) | 2.8 (1.0) | 3.0 (1.2) |
| Family history of eczema | 1270 | 514 (40.5%) | 251 (39.5%) | 263 (41.5%) | 372 (44%) | 142 (34%) |
| Family history of any atopic disease ^a^ | 1271 | 1049 (82.5%) | 529 (83.2%) | 520 (81.9%) | 721 (84%) | 328 (79%) |
| Both parents have an atopic disease ^a^ | 1269 | 386 (30.4%) | 192 (30.2%) | 194 (30.6%) | 273 (32%) | 113 (27%) |
| Smoker living in the house during pregnancy | 1269 | 222 (17.5%) | 105 (16.5%) | 117 (18.5%) | 121 (14%) | 101 (24%) |

^a^ Any of eczema, hay fever, asthma. BCG: bacille Calmette-Guérin.

Categorical variables are reported as number (%), continuous variables are reported as mean (interquartile range).

###

### **Table E2**: Cumulative incidence of eczema in the first five years following randomisation, using multiple imputation and complete case analysis

|  | Multiple imputation | | Adjusted risk difference | Complete case analyses | | Adjusted risk difference |
| --- | --- | --- | --- | --- | --- | --- |
|  | BCG | Control | (95% CI)^a^ | BCG | Control | (95% CI)^a^ |
| **Primary analysis** (William’s UK diagnostic tool) |  |  |  |  |  |  |
| Eczema (ever) in the first 5y of age | 37.1% | 45.3% | -8.2 (-14.1, -2.2) | 187/460 (40.7%) | 207/394 (52.5%) | -11.8 (-18.5, -5.2) |
| Sensitivity analysis (excluding the catch-up questionnaire data) | 40.2% | 49.5% | -9.3 (-16.6, -2.1) | 187/403 (46.4%) | 207/352 (58.8%) | -12.4 (-19.4, -5.3) |
| **Secondary analyses** (alternative measures of eczema) |  |  |  |  |  |  |
| Parent report of medically diagnosed eczema | 41.1% | 45.2% | -4.0 (-10.3, 2.4) | 229/502 (45.6%) | 224/431 (52.0%) | -6.3 (-12.7, 0.1) |
| Extended definition of eczema (UK diagnostic tool or medically diagnosed) | 50.5% | 57.8% | -7.4 (-13.9, -0.8) | 270/492 (54.9%) | 283/425 (66.6%) | -11.7 (-18.0, -5.4) |
| Use of topical steroid | 64.8% | 72.8% | -8.0 (-14.9, -1.2) | 272/397 (68.5%) | 277/370 (74.9%) | -6.3 (-12.7, 0.0) |
| Clinically significant (UK diagnostic tool and medically diagnosed and steroid use) | 24.5% | 26.4% | -1.9 (-7.2, 3.3) | 131/461 (28.4%) | 128/386 (33.2%) | -4.7 (-10.9, 1.6) |
| **Subgroup analyses** |  |  |  |  |  |  |
| Female | 36.4% | 41.9% | -5.7 (-14.2, 2.9) |  |  |  |
| Male | 37.7% | 48.5% | -10.9 (-19.5, -2.2) |  |  |  |
| Both parents are atopic | 43.0% | 54.4% | -11.6 (-22.7, -0.5) |  |  |  |
| None or one parent is atopic | 34.4% | 41.2% | -6.8 (-14.0, 0.3) |  |  |  |
| Mother BCG naïve | 34.1% | 43.3% | -9.3 (-16.5, -2.0) |  |  |  |
| Mother previously BCG vaccinated | 46.1% | 47.1% | -0.9 (-12.8, 11.0) |  |  |  |

^a^ adjusted for stratification factor of mode of delivery.

BCG: bacille Calmette-Guérin; CI: confidence interval; IQR (interquartile range).

### **Figure E1**: CONSORT diagram.


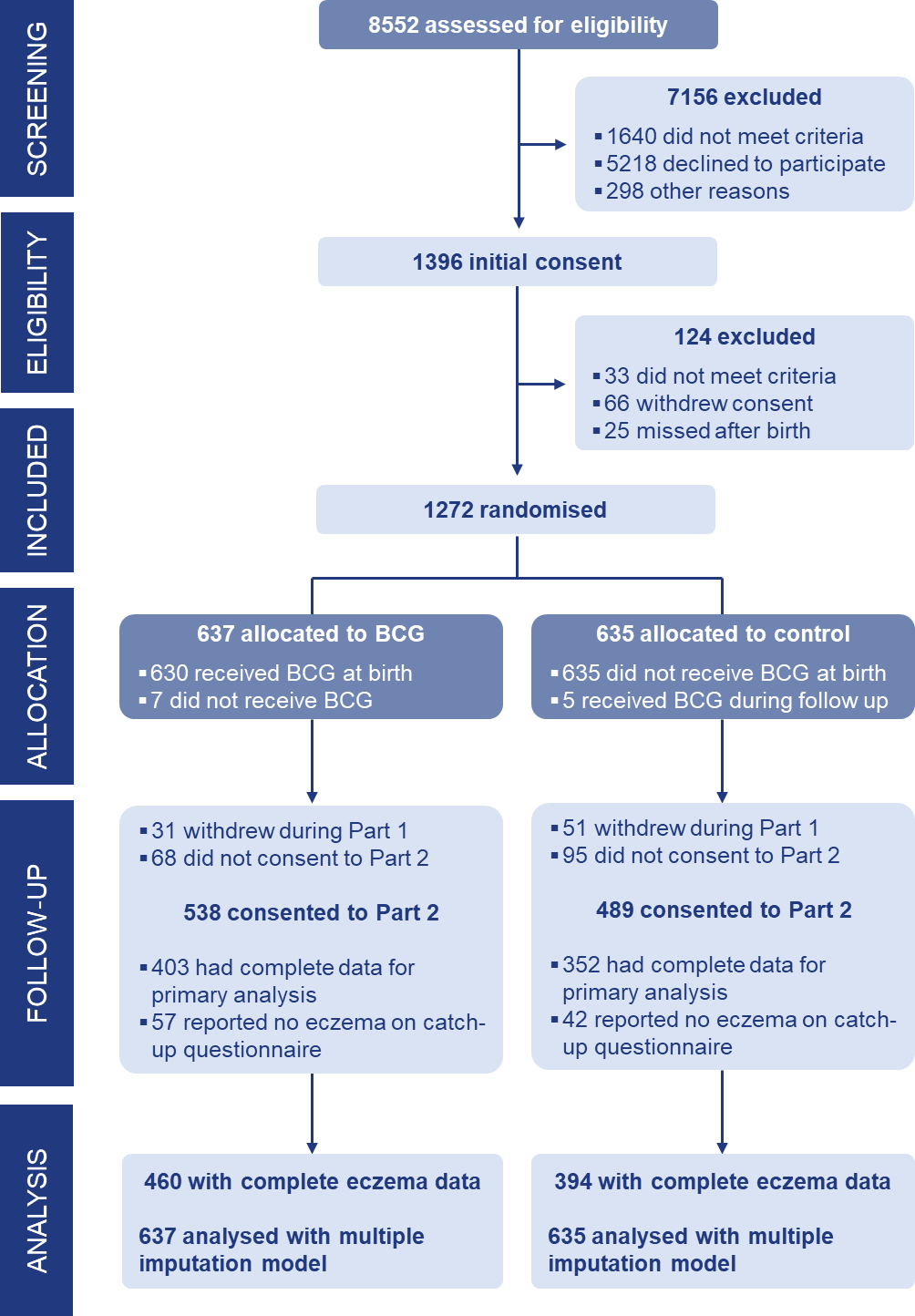


BCG: bacille Calmette-Guérin.

**Figure E2**: Subgroup analyses of the cumulative incidence of eczema at 5 years of age.


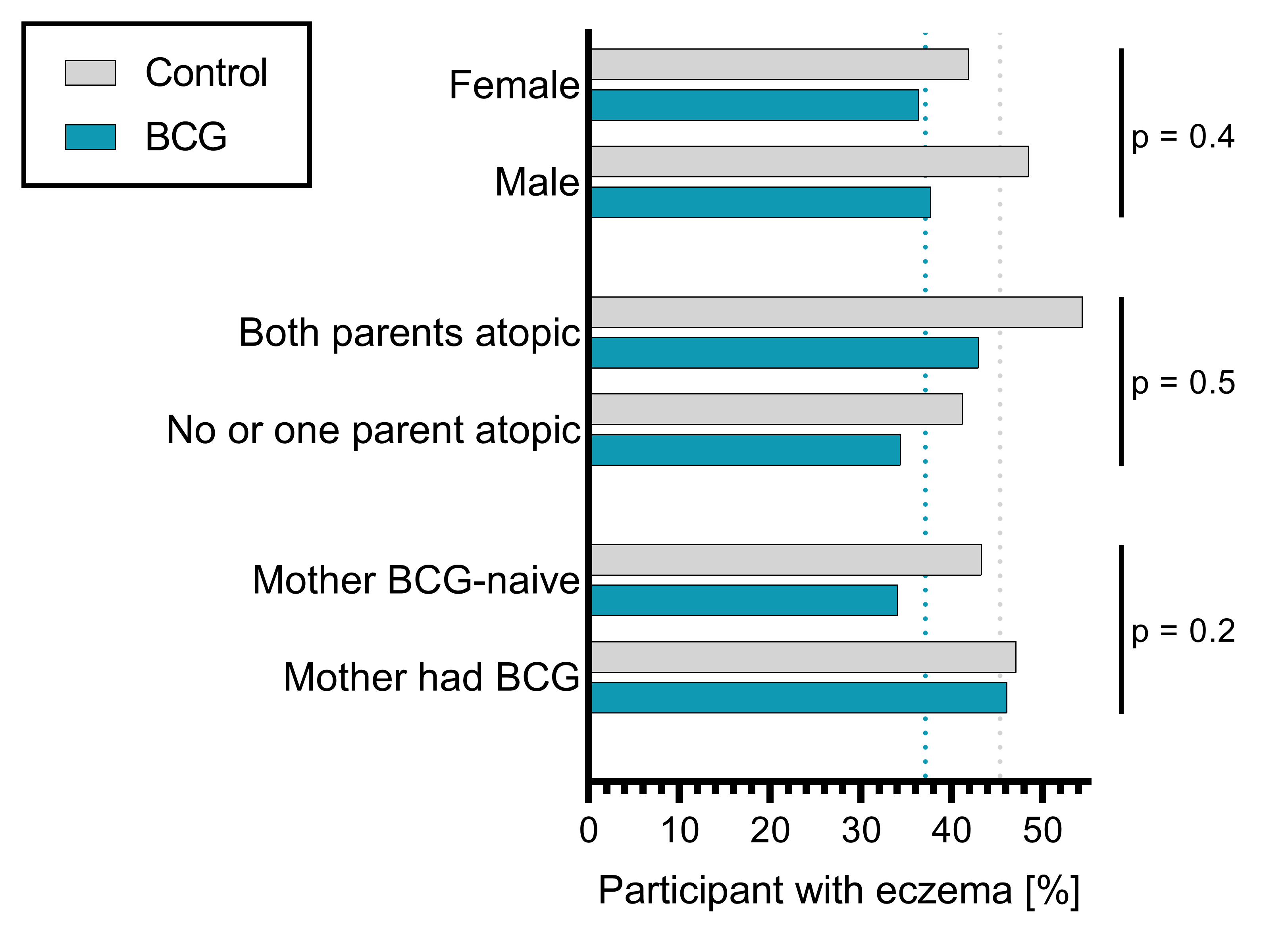


Bars represent the proportion of participants fulfilling the criteria for the primary analysis of eczema in prespecified subgroups. The vertical dotted lines represent the proportion of participant with eczema in the whole BCG (blue) and control (grey) groups. P-values are for the interaction term (BCG status by variable).

**Figure E3**: Survival curve of eczema onset in the first five years following randomisation, complete case analysis


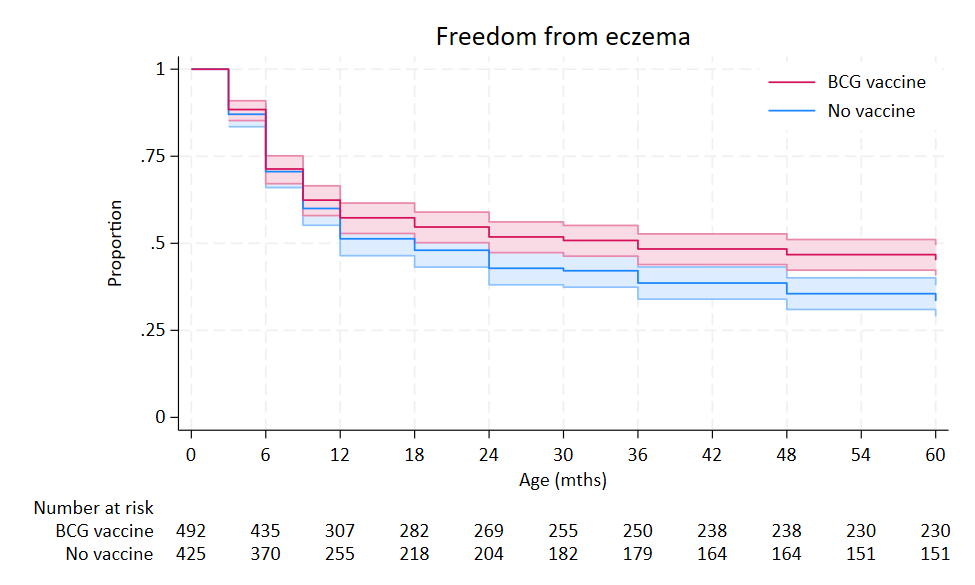


Log-rank test p-value=0.002. Age of onset of eczema was defined, among participants fulfilling the extended definition of eczema, as the first time point (i.e. the first questionnaire) when the parents reported a medical diagnosis of eczema or the participant fulfilled William’s UK diagnostic criteria. Questionnaires were sent at 3, 6, 9, 12, 18, 24, 30, 36, 48 and 60 months of age.
